# A Global Review of the Woody Invasive Alien Species *Mimosa pigra* (Giant Sensitive Plant): Its Biology and Management Implications

**DOI:** 10.3390/plants11182366

**Published:** 2022-09-10

**Authors:** Amali Welgama, Singarayer Florentine, Jason Roberts

**Affiliations:** Future Regions Research Centre, Institute of Innovation, Science and Sustainability, Federation University Australia, Ballarat, VIC 3350, Australia

**Keywords:** eradication, invasive weeds, management strategies, plant biology, plant invasion

## Abstract

Populations of invasive alien plants create disruptive plant communities that are extremely adaptable, imposing severe ecological impacts on agriculture, biodiversity and human activities. To minimise these impacts, prevention and effective weed management strategies are urgently required, including the identification of satellite populations before they invade new areas. This is a critical element that allows weed management practices to become both successful and cost-effective. *Mimosa pigra* L. (Giant sensitive plant) is an invasive weed that has spread across various environments around the world and is considered one of the world’s top 100 most invasive plant species. Being adaptable to a wide range of soil types, in addition to its woody protective prickles and low palatability, *M. pigra* has quickly spread and established itself in a range of habitats. Current control methods of this species include biological, chemical and physical methods, together with attempts of integrated application. Reports suggest that integrated management appears to be the most effective means of controlling *M. pigra* since the use of any single method has not yet proved suitable. In this regard, this review synthesises and explores the available global literature and current research gaps relating to the biology, distribution, impacts and management of *M. pigra*. The contribution of this work will help guide land managers to design appropriate and sustainable management programs to control *M. pigra*.

## 1. Introduction

*Mimosa pigra* L. (Giant sensitive plant) is an erect, prickly shrub or small tree, which is native to Tropical America [1]. Outside of its native regions, *M. pigra* has been reported to cause significant economic and environmental impacts across various environments when appropriate long-term management is not implemented [2,3,4,5]. If left untreated, the species can quickly form dense, monospecific, leguminous stands that can spread over thousands of hectares [2,3,4,5]. As a result, *M. pigra* has been listed as one of the top one hundred most invasive plant species in the world and is of particular concern within Africa, Australia and Southeast Asia [5,6,7,8,9,10,11]. *Mimosa pigra* is commonly found growing along floodplains, irrigated landscapes, seasonally wet savannas and waterways where it strongly competes against native and pastoral species for resources such as light, water and soil nutrients [5,7,12,13]. Dense *M. pigra* infestations have also been known to significantly impact biodiversity, cropping systems and livestock production as they (i) can smother native and pastoral species, (ii) contain allelopathic properties that suppress the growth of adjacently growing species, (iii) have an aggressive and quick growth habit and (iv) limit water accessibility to livestock and people [5,14,15,16,17]. In addition, *M. pigra* is capable of doubling its population size within 1.2 years when growing adjacent to a river system, although isolated populations away from large water sources may take up to 6.7 years to double in size [6]. In this regard, it is clear that to overcome the threat imposed by this invasive woody shrub, strict control measures need to be rigorously implemented.

Current management practices used to control *M. pigra* around the world include biological, chemical and physical strategies, and in some countries, including Australia and Zambia, integrated practices are also being utilised [5,13,18,19]. Although some of these strategies appear promising in the control of *M. pigra*, many fail as a single-use application and require an ongoing or integrated approach over several years, which can be costly and time consuming [5,20]. In this regard, detailed collection and exploration of information relating to the characteristics of *M. pigra* are urgently required to help land managers design appropriate and sustainable management programs to control the species. To contribute to this work, this review will synthesise and explore the available global literature and existing research gaps on the biology, distribution, impacts and management of *M. pigra*.

## 2. Methodology

This review was conducted between November 2021 and September 2022 by exploring the global English literature that directly relates to the biology, impacts and management of *M. pigra*. The literature search for this review was conducted using Google Scholar by searching the terms ‘*Mimosa pigra*’ or ‘Giant sensitive plant’ plus one of the following terms: Biology, distribution, impacts, invasive, management or weeds. Each paper identified during this process that had these terms either in their title, abstract or presented as a keyword was then selected and scanned for their suitability for this review.

## 3. Taxonomy

*Mimosa pigra* L. belongs to the genus *Mimosa* (Fabaceae), which contains between 400 to 500 species [21]. There are currently three varieties of *M. pigra* found globally, which include *M. pigra* var. *asperata* L., *M. pigra* var. *dehiscens* Barneby and *M. pigra* var. *pigra* L. [22,23,24]. Research has shown that there is often confusion in identifying the differences between these varieties due to their similar appearance and biology [22,23,24]. In this regard, this review will explore the species in a broad sense due to the limitations in the existing literature on the different varieties. With this in mind, future research to fill in this knowledge gap and explore the different biological characteristics of these varieties would be of value. The value of this information would be useful in identifying genetic variation between populations and any potential areas that may be at risk of future invasion from *M. pigra*.

## 4. Distribution

**Figure 1:**
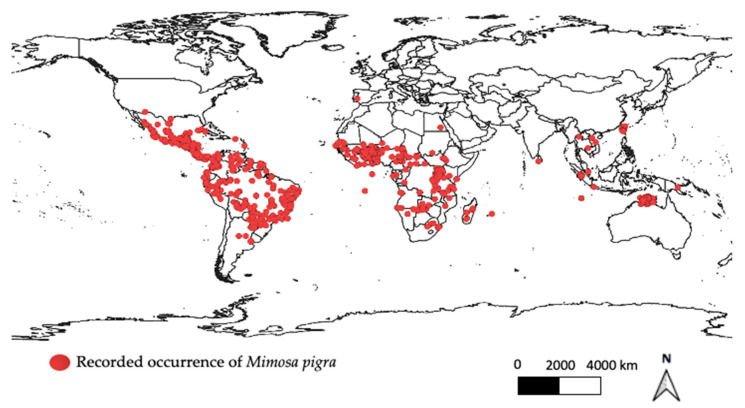
Global distribution of *Mimosa pigra* L. [36] (DOI: 10.15468/dl.d75f66).

*Mimosa pigra* is native to Tropical America and is found across Argentina, Belize, Bolivia, Brazil, Chile, Columbia, Costa Rica, Cuba, Ecuador, El Salvador, French Guiana, Guatemala, Guyana, Mexico, Nicaragua, Panama, Paraguay, Peru, the Republic of Honduras, the Republic of Trinidad and Tobago, Uruguay and Venezuela [1,3,25,26,27]. Research by Sheded and Hassan [28] also described *M. pigra* as an endangered shrub in Egypt, suggesting that it is also native within this region. It has also been reported that *M. pigra* var. *asperata* and *M. pigra* var. *dehiscens* are only locally found within the species’ native regions in Brazil, Belize, Columbia, Cuba, Mexico, Nicaragua and Paraguay [1,23,29,30,31]. On the other hand, *M. pigra* var. *pigra* is much more widespread and is commonly found outside of its native region, being found on the continents of Africa, Asia, Australia, Europe, North America and South America (Figure 1) [1,23,31,32,33]. It is reported that *M. pigra* was purposely introduced into many of these regions as a cover crop, ornamental or green manure plant [32,33].

In Africa, *M. pigra* can be found in Angola, Benin, Botswana, Burkina Faso, Burundi, Cameroon, Central African Republic, Chad, Egypt, Ethiopia, Gambia, Ghana, Guinea Bissau, Guinea, Ivory Coast, Kenya, Liberia, Malawi, Mali, Mauritania, Mozambique, Namibia, Niger, Nigeria, Rwanda, Senegal, Sierra Leone, Somalia, South Africa, Sudan, Tanzania, Togo, Uganda, Zambia and Zimbabwe, but is only described as invasive in some of these countries [11,19]. This wide distribution indicates the species’ ability to adapt to the warm African environment, as opposed to the tropical environments where it is found within its native regions [11,19]. In particular, *M. pigra* is classified as a category-three invader in South Africa, therefore the propagation of this plant is prohibited unless special permission is granted by state law [10,11]. It can also be found on the islands of Madagascar and Mauritius [10,11]. *Mimosa pigra* has also been introduced into Asia, being found in Cambodia, India, Indonesia, Laos, Malaysia, Myanmar, Sri Lanka, Thailand and Vietnam, where it is now recognised as a widespread invasive weed [11]. It is noted that this species was introduced into Thailand as a green manure plant and a cover crop in 1947 and was subsequently taken to Malaysia and nearby countries as a cure for snake bites [34]. In Australia, *M. pigra* was introduced sometime before 1891 as a seed contaminant or as a curiosity plant because of its sensitivity to human touch [35]. The plant was regarded as occasionally problematic until the late 1950s, whereupon its shift to open flood plains started to produce challenging monospecific stands [35]. Currently, in Australia, *M. pigra* is abundant in the Northern Territory, the flood plains of Adelaide, and the Daly, Finniss, Mary and East Alligator River systems and is listed as a noxious species across the country [10,11]. Small infestations have also been observed in Western Australia and Queensland, where the plants have already been subject to eradication activities [19].

## 5. Habitat

In its native region, *M. pigra* can be found either as a single plant or in thickets, but as a successful invader in other tropical regions of the world, it predominantly occurs as robust thickets, particularly in disturbed lands where there is abundant water [7]. Tropical climates with distinct wet and dry seasons are favoured for its growth, and regions with less than 750 mm of rainfall are not expected to provide suitable invasion sites, with the exception of areas directly around water bodies [25]. In rainforests where the typical rainfall is above 2250 mm, *M. pigra* establishment is relatively unlikely due to the prevailing high level of existing plant competition, and in the cooler subtropics, growth has been observed to be shorter and less aggressive compared to growth in tropical areas [7]. With regard to supporting growth media, this woody shrub can thrive in a range of soil types including heavy black cracking clays, sandy clays and coarse siliceous river sand [7,37]. In Australia, high seed production and greater life expectancy are observed when the species becomes established in black cracking clays, whilst high seed longevity is observed in sandy clays [7,37], but some variations across invasion sites have been noted [26].

## 6. Plant Morphology and Characteristics

*Mimosa pigra* can grow up to four to six meters in height and create dense stands with an average density of one plant per meter squared [27,38]. The stems contain long broad-based prickles, which are approximately 7 mm in length [7]. Its leaves are bipinnate and edged with a setulose margin that has a parallel and mid-ribbed venation [7]. These leaves are also sensitive to touch through the pinnules, pinna rachises and the petiole [7]. The flowering period of *M. pigra* occurs mostly in late Spring to Autumn (Table 1), where it produces thousands of pink flowers, which are 2 cm in diameter and contain approximately 100 flowers per axillary head [27,39]. Upon successful pollination, each flower head can produce up to seven pods containing 21 seeds [27]. This process of ripened seed production from the flower buds typically takes five weeks [7], with the ripe seeds being oblong and brown to olive green in colour [7]. Differences in leaf and pod morphology are evident depending on the country it is found within and seasonal weather variation, with broader pods being observed in Thailand compared with comparatively slender pods found in Australia [7]. To aid dispersion, the hirsute pods break into single-seeded segments that are partially dehiscent, allowing them to remain afloat for an infinite period [38]. Research has also shown that plant morphology can differ when the species is under stress from abiotic and biotic factors [40]. Research by NurZhafarina and Asyraf [40] highlighted that *M. pigra* shows high morphological variation when faced with intraspecific competition. In fact, habitats with a high species density often result in *M. pigra* growing taller and producing less viable seeds, whereas a habitat with low species diversity results in *M. pigra* becoming sturdier and producing more viable seeds [40]. This suggests that competition can significantly influence the species’ morphology and overall growth and competitive performance [40,41].

*Mimosa pigra* is a common invader in wetlands and flooded areas because it can produce adventitious roots near the soil surface as a defence against anaerobic waterlogged conditions [42], but it can also resist drought conditions, which increases its invasive ability [6]. Having a low nutrient requirement, *M. pigra* can grow in a wide range of soil types including sand, red and yellow alluvial soils, silty loams and heavy black cracking clays [43]. The average growth rate of *M. pigra* grown under optimum conditions is 1 cm per day and is predicted to double its presence in an infested area within a year [6]. Its rapid plant maturity and seed production during the first year of growth contribute to its invasive potential [38]. Depending on the environmental and growth conditions, the average seed production rate per year is estimated to range between 9000 and 12,000 seeds per meter squared [37], but plants with the highest productivity grown in the field are observed to produce over 220,000 seeds per year [44]. The longevity of *M. pigra* seeds within the soil can extend up to 23 years, although this is highly dependent and variable on the soil type and depth of seed burial [4]. The bristled seedpods can assist the seeds’ dispersion over long distances by becoming attached to animals or humans, with seedpods also commonly transported by moving water bodies [42], where the buoyant pods are supported by the surface tension of water [7]. Other dispersal methods include seeds entrapped in soil or mud particles, which adhere to agricultural vehicles [38], and grazing animals passing dung containing *M. pigra* seeds [35]. The plant is capable of resprouting from remaining stumps after severe pruning [45]. It is also estimated that 90% of mature *M. pigra* plants and 50% of seedlings can regrow if exposed to moderate fire events [4]. This emphasises the need for ongoing and repeated management of the species as one control event such as fire may not be suitable. It would also be of value for future research to examine the seed germination requirements of *M. pigra* from a range of populations from different climatic regions. This information would be of value to land managers by allowing them to understand which factors facilitate the germination of *M. pigra*. This information would also help to guide them in making suitable and confident decisions regarding the control of the species in its early stage of development.

## 7. Environmental and Social Impact

Due to its invasive character, *M. pigra* poses a huge problem for the conservation of tropical ecosystems. Once it is established in the landscape, it becomes the dominant species and prevents the establishment of other species within the understory [2]. This dominance can severely alter the vegetation and structure of floodplains and swamps within the region [2]. These aggressive populations of *M. pigra* out-compete native herbaceous plants for light, moisture and nutrients, whilst dense stands grown under native tree canopies can also prevent the seedling establishment of these trees by limiting essential light penetration [2,4]. It has also been reported that *M. pigra* contains allelopathic properties including the phytotoxin mimosine, in addition to other phenolic, tannin and flavonoid compounds [13,14]. These compounds found within *M. pigra* cause inhibitory effects to adjacently growing vegetation, ultimately giving *M. pigra* a competitive advantage. Alternatively, research has suggested that these compounds could be utilised and manipulated as an aqueous solution and applied to control various other weed species such as *Echinochloa crus-galli* L. (Barnyard grass) and *Lolium multiflorum* L. (Italian ryegrass) [14,46]. Although this is suggested, further research is required to investigate these phytotoxic compounds produced by *M. pigra* on a range of native species. These allelopathic properties could also explain the successful invasion of *M. pigra* across various environments around the world. The altered floral diversity and hydrology caused by *M. pigra*-dominant areas also affect the living conditions for native fauna, and it has already been noted that losses of habitat, breeding sites and fruit trees have been negatively related to the number of native fauna in many *M. pigra*-affected areas [2,4].

*Mimosa pigra* not only affects the biodiversity of an area but is also seen to impact the socio-economy of a community [4,5,42]. Day-to-day human recreational activities and tourism opportunities that are dependent on accessible water bodies, in addition to agricultural requirements such as available drinking water for cattle and irrigation for crops, are greatly threatened by *M. pigra* invasion [4]. Dense stands of *M. pigra* can block roads and pathways, which can limit accessibility to croplands, water bodies and grazing areas [5,42]. Grazing animals rarely feed on *M. pigra,* and as a result, this contributes to its uncontrolled growth and spread into new areas [5,42]. It has even been reported that the establishment of this invasive species has significantly reduced the available grazing land in Zambia, and as a result, milk and livestock production has been heavily impacted [5]. Consequently, the disruption to livestock production in these communities has even contributed to significant economic loss, illness and increased death rates [4,5,42]. In Africa, the seasonal floodplains have traditionally provided many communities with essential services including fishing, seasonal cropping, renewable fuelwood supplies and rich grazing for livestock [5,47]. These services are expected to be heavily threatened by the increasing invasion of *M. pigra* [5,47], impacts that have also been evident in Australia [2], Cambodia [3,40] and Vietnam [48], which emphasises the extent and distribution of this problem.

## 8. Control Measures

Whilst controlling *M. pigra* is usually focused on dealing with highly infested areas, it is also recommended that management activities should also be centred around isolated or smaller populations [49]. Such activities will help to prevent the establishment of dense monocultures and reduce the future costs associated with its management. Although this is recommended, it is still critically important to control densely infested areas as they can be a large source point for new seeds, which are known to be long-lived and can remain viable for up to 23 years [4,26]. Whilst attempts to control *M. pigra* infestations have been centred on chemical and physical control approaches, the use of biological control has also shown promising signs in controlling the species [19,50]. In conjunction with these approaches, managers have also been searching for possible native plant species to create strong competitive interactions with *M. pigra* to reduce and suppress its growth [51,52]. It has also been suggested that vector control should be introduced to track and eliminate *M. pigra* seed dispersal as a method of prevention. Hence it is generally accepted that integrating existing control measures will result in greater efficiencies [13,20,53,54], and in Table 2, Table 3 and Table 4, the most commonly used control measures that can be conducted at different growth stages of *M. pigra* are shown.

### 8.1. Physical Control

As reported in the study of Cook et al. [50], cutting, hand-pulling and burning can be usefully implemented as a physical control measure to control incipient outbreaks of *M. pigra* (Table 2). In the case of larger infestations, bulldozing, chaining and ploughing can be used [58], although native species and soil conditions may become altered by these actions, which should be considered. Notwithstanding the success of these physical methods, the implementation of follow-up control measures is strongly advised due to the regenerative success of produced fragments [72]. Moderate burning has been observed to be ineffective with green *M. pigra* plants, and if such a stand is subject to fire, it can regenerate from the bud regrowth at the stem base. In addition, mild fire is a seed germination stimulator for *M. pigra* seeds, and hence burning can enhance seed germination [59]. However, direct application of gelled gasoline, or in dense monospecific stands, aerial application of this intense-burning fuel, has been reported to result in the destruction of *M. pigra* [59]. It has been suggested that when conducting planned burning on *M. pigra*, the season of burning is a critical factor, as it has been shown that immediate floods after burning are favourable for preventing *M. pigra* regeneration [20]. If some leaves remain above the water level, and if the plant or plant remnants after a fire are fully submerged, they are drowned within three months [73]. If, however, the time between the burn and the flood occurrence allows the *M. pigra* plants to establish and grow beyond the heights of flooding waters, follow-up treatments will be required to assure successful control [59].

### 8.2. Chemical Control

The primary method of controlling *M. pigra* is with the use of herbicides, and in Australia, Malaysia and Thailand, large numbers of herbicides have been tested against *M. pigra*, with many being effective [7,13,42,43,48,74,75,76]. In Australia, more than 21 herbicides, representing different application strategies including spraying and stem injections together with soil application, have been tested [7]. Among those herbicides, 2,4,5-T, tebuthiuron, fluroxypyr, metsulfuron-methyl (74223-64-6), dicamba, glyphosate and hexazinone have been previously used [55]. Aerial herbicide spraying can also be reasonably effective when conducted in the wet season, but reports suggest that it might not result in 100% plant mortality [72]. In addition, large-scale herbicide application warrants careful consideration as it may contribute to further environmental pollution, especially near waterways and native species. In this regard, future research on the management of *M. pigra* should consider integrating a range of techniques, such as biological control, burning, herbicide application or manual removal to minimise chemical exposure to the environment and provide more confident control [20].

Applying dicamba, glyphosate, hexazinone, imazapyr, triclopyr, triclopyr + picloram and triclopyr + picloram + 2,4-D to the cut stumps of severed *M. pigra*, has also shown success in controlling *M. pigra* [56]. 2,4-D was the primary herbicide used in the 1960s and 1970s but required repeated applications to combat new regrowth [56]. When public health concerns arose in the mid-1980s [42], new herbicide options were explored with different rates and application methods; however, it is important to note that the effectiveness of control is known to be highly dependent on the season of application [56]. Dry-season application inevitably results in more regrowth, and thus higher concentrations are required for satisfactory control, but it has been observed that dicamba and hexazinone are highly effective on cut stump applications during both dry and wet seasons [7]. Targeting the plant’s active growing season will also enhance herbicide uptake and efficacy, resulting in better control of the weed. For basal bark herbicide applications, triclopyr, triclopyr + picloram, dicamba and 2,4,5-T plus picloram, either as a diesel mix or in an aqueous solution, are recognised as potential herbicides [13]. Compared to the basal bark herbicide application, reduced efficacy was observed when herbicides were applied as stem injections into *M. pigra* [7]. It is also important to note here that cut and herbicide application methods can be costly and time-consuming for large infestations [15,48], therefore should only be considered in smaller or isolated populations. Additionally, it is also likely that seeds will regenerate from the seedbank following the removal of mature plants, therefore follow-up applications of either herbicide application or manual removal would be necessary.

When herbicide control measures were first implemented in the Northern Territory in Australia, 2,4,5-T was applied as a foliar spray in a mix of either water or diesel [42]. Picloram plus 2,4,5-T mixed with diesel was also used as a basal bark spray or as a foliar application [42,77], and in 1980, glyphosate, delivered by a high-volume foliar sprayer, was introduced for control in town areas [42]. The residual herbicide hexazinone was also used as a soil application to reduce the emergence of *M. pigra* [25]. With respect to herbicide treatments in general, application time is regarded to be critical. In Australia, 2,4,5-T and tebuthiuron are traditionally applied before the floodplains are inundated. When this application defoliates the *M. pigra* plants, fluroxypyr is then applied to any surviving plants [78]. In Thailand, bromacil or bromacil + diuron is recommended as an application in non-agricultural lands and on dam walls, while Fosamine ammonium (25954-13-6) is used for roadsides, alongside canals and in water reservoirs as a foliar spray [7]. Foliar application of dicamba has also been recommended for non-agricultural lands, water canals with a water depth greater than 1 m and roadsides. Glyphosate is also recommended for application in all the above-mentioned *M. pigra* habitats, with necessary precautions taken when applying near water bodies. Glyphosate can also be used in agricultural lands before cropping takes place or after harvesting the crops [7].

Tebuthiuron is a residual herbicide that is absorbed through the roots, and hence, it is advised to apply this compound while the plant is in its actively growing phase [7,61]. According to the study by Lane [61], tebuthiuron has not been effective on *M. pigra* seedlings, evidencing a percentage survival rate of 43%. Fluroxypyr and metsulfuron-methyl are recommended to be used as aerial applications, with the large dense stands of *M. pigra* most likely to require the use of an aircraft to gain sufficient access [79]. The efficacy of fluroxypyr is evident in the study of Paynter and Flanagan [20], where its application resulted in significant control, and, in addition, its selective action on dicotyledons allowed monocotyledon species to compete favourably with any seedling survivors. Given the mediocre effectiveness of some herbicides and the aggressive nature of *M. pigra*, it is recommended that the infested area should be subjected to intense fire after the chemical treatment to minimise any regrowth from the remaining plants [79].

### 8.3. Biological Control

Numerous natural enemies for *M. pigra* have been identified in its native range [62]. As with other attempts at biological control, significant attention has been given to each attacking agent’s host specificity, and even in the face of significant evidence for a species’ ability to attack and damage *M. pigra* plants, there must be compelling information regarding the unlikelihood of the agent to affect other vegetation before it is introduced and released into a new environment [62]. Once introduced, careful monitoring of the survival, distribution and abundance of the biological agent is a critical factor in the evaluation phase of biological weed control [64,80,81]. Notwithstanding these concerns, due to the high costs related to chemical and physical control of *M. pigra*, biological control is widely regarded as providing the most effective long-term control strategy in Australia [42,67].

The first exploration of natural enemies against *M. pigra* was conducted in 1980 in Brazil [19]. There have been many introductions of agents since this time due to this strategy’s promising potential for controlling established stands [67]. Significant reduction of the *M. pigra* seed bank under thick plant cover [20,63,78,82] and a noticeable decline in density as a result of action by biological species have been observed [27]. According to these studies, *Carmenta mimosa* was identified as the most damaging biological agent for *M. pigra* [20,53,63], where evidence of the reduced seed production [20] and areas of canopy opening up caused by the high densities of *C. mimosa* has reduced the competitiveness of *M. pigra* stands with other vegetation, especially at the stand edges [63].

The first insects introduced for *M. pigra* biological control in Australia were *Acanthoscelides quadridentatus* and *A. puniceus*; these are Mexican seed-feeding beetles [40]. They were released in 1983 and 1984 in Australia and Thailand, respectively [40]. In addition, *Chlamisus* sp., which feeds on the leaf and bark of *M. pigra,* was introduced from Brazil and released in Australia and Thailand in 1985 [1]. The stem-boring moths, *Neurostrota gunniella* and *Carmenta mimosa,* were also released [7], and since then, several other biological agents for *M. pigra* have been introduced. The list includes the beetle species *Acanthoscelides puniceus*, *Chlamisus mimosae*, *Malacorhinus irregularis* and *Coelocephalapion pigrae*, and the moth species *Neurostrota gunniella*, *Carmenta mimosa* and *Macaria pallidata* [36,83]. It is noted that fungi such as *Diabole cubensis* and *Phloeospora mimosae-pigrae* and beetles such as *Acanthoscelides quadridentatus*, *Coelocephalapion aculeatum*, *Chalcodermus serripes* and *Sibinia fastigiata* have also been introduced as biological agents for *M. pigra* but, at this time, they have not succeeded in becoming established [20] (Table 4). It is also noted that future research on the biological control of *M. pigra* should consider integrating other control strategies such as burning, herbicide application and physical control for more confident control. Research has suggested that integrating a range of methods along with biological control helps to improve success [54], although, in the case of *M. pigra*, this requires further research to discover which combinations are most efficient.

## 9. Future Management Considerations

As emphasised above, *M. pigra* poses a significant impact on biodiversity and human socio-economic activities. In terms of control, addressing small, isolated outlier populations at their earliest detection and implementing integrated management strategies is currently suggested to be the most effective approach to controlling this woody shrub. In concert with these actions, given the high invasiveness of the species and its seed viability over a long period, continuous monitoring of any treated site is advisable. Of importance is the observation that *M. pigra* is susceptible to grass competition [72]. In this regard, planting native grasses or creating competitive pastures in areas at risk of invasion by *M. pigra* could be a viable option for suppressing its growth. For dense *M. pigra* stands, aerial herbicide application will open up the canopy, allowing competing herbaceous plants to grow. Intense fire can then be used to clear the area, followed by the introduction of competitive pasture species into the area. Even though the seed germination of *M. pigra* is known to be stimulated by fire, seedling growth will be suppressed by the growth of competitive pasture seedlings [72]. Implementing biological control measures has shown significantly promising results compared to other control measures, but notwithstanding these control measures, further research needs to be carried out with simulated abiotic and biotic environmental stressors to identify their influence on *M. pigra* growth and establishment [84,85]. Such studies will provide valuable information related to the optimum conditions for growth, and this will lead to new effective management practices since continuous, dynamic and focused management is required for the mitigation of impacts associated with *M. pigra* invasions. Despite the advances in management practices and awareness, *M. pigra* continues to remain a globally invasive species, and extensive and more recent research and control experiments need to continue to be conducted in order to suppress the impact caused by this tropical woody shrub. It is also noted that early detection protocols and the identification of isolated *M. pigra* populations are critical steps for assisting and planning the successful long-term control of the species [21]. This could be achieved using drone technology for mapping and identifying difficult-to-reach areas that are infested with *M. pigra*.

## 10. Conclusions

This review highlights that an integrated and long-term management approach is highly necessary to control *M. pigra* and reduce its economic, environmental and social impact. Due to this species commonly being found close to water bodies or in difficult-to-access terrain, a significant financial and laborious investment is often required. However, this can be reduced if small, isolated populations are identified and immediately controlled before they form dense monocultures. Future lines of research should aim to focus on a greater understanding of the life cycle and susceptible growth stages of *M. pigra* since this is not yet at a satisfactory level. This could be achieved by a further investigation into the biology of the species across a range of climatic and environmental conditions. Such a level of detail will allow for greater confidence when designing long-term control approaches for the species at both localised and landscape scales. Given the scarcity of the available relevant global literature, this review is anticipated to provide the first step for future studies toward building a more comprehensive global *M. pigra* control schedule.

## Figures and Tables

**Table 1:** Average life cycle of *Mimosa pigra* throughout each season.

Plant Life Cycle	Summer			Autumn			Winter			Spring		
Seed Germination												
Flowering												
Podding												
Active Growth Period												

Note: Shading represents the seasons that have been reported in the literature where life cycle events of *M. pigra* have been observed. These conditions are likely to vary according to geographical location or climate variation [1,7,27,39].

**Table 2:** Commonly used physical control measures implemented at different growth stages of *Mimosa pigra.*

Treatment	Growth Stage				Reference
	Seeds	Seedlings	Vegetative Stage	Reproductive Stage	
**Hand pulling**	N/A	Complete control can be achieved in the early stages when all the plant parts are removed.		Less successful and costly.	[48,55,56]
**Stem cutting**	N/A	N/A	Should be done before flooding to drown the new regrowth. Plant age and level of plant cover affect the cost.		[48]
**Ploughing**	N/A	Ploughing uproots the whole plant or the remaining root parts resulting from stem cuts thus preventing regrowth. Provides better seedbed establishment for pasture.			[55,57,58]
**Stick raking**	N/A	Equipment attached to a bulldozer or tractor removes the entire stump and root system with minimal soil disturbance.			[55]
**Chaining**	N/A	N/A	A heavy chain is pulled between two bulldozers, physically removing mature plants. Suitable for use during the wet season for dense infestations.		[55,58]
**Chopper rolling**	N/A	A dense drum equipped with blades is pulled behind a tractor, which knocks down and macerates the plants.			[55]
**Burning**	Can either destroy or stimulate seed germination, therefore should be followed by herbicide treatments.	N/A	Difficult to burn when green.	Burnt plants can regrow from buds at the stem base.	[20,57,59]

**Table 3:** Commonly used chemical control measures implemented, mainly in Australia, at different growth stages of *Mimosa pigra.*

Active Ingredient	Growth stage				Reference
	Seeds	Seedling	Vegetative Stage	Reproductive Stage	
**Glyphosate**	N/A	Effective at all growth stages and used in cut stump application mixed with water.			[55]
**Triclopyr butoxyethyl ester**	N/A	Effective when plants are young.			[48]
**Metsulfuron methyl**	N/A	Most effective when plants are young and when used as aerial control for infestations.			[55]
**Glufosinate ammonium**	N/A	Should be applied when the plant is actively growing. Foliage should be covered thoroughly.			[60]
**Tebuthiuron**	N/A	Should be applied before seed set when the plant is actively growing. Can be used as both a soil and aerial application. Higher rates are required on dense growth or heavy clay soils.			[1,7,61]
**Fluroxypyr**	N/A	Foliar application when actively growing and basal bark or cut stump application when mixed with diesel.			[20,53,55]
**Dicamba**	N/A	Aerial control when infested and foliar application for actively growing plants.			[55]
**Hexazinone**	N/A	Not recommended for continuous use in large areas.			[55]

**Table 4:** Commonly used biological control measures implemented at different growth stages of *Mimosa pigra.*

Treatment	Growth Stage				Reference
	Seeds	Seedling	Vegetative Stage	Reproductive Stage	
***Carmenta mimosa* (Sesiidae—Lepidoptera)**	N/A	Attacks large stems but is effective on new shoots and young plants. Results in dead and broken branches; also reduces seed rain and hence a decline in the seed bank.			[36,48,62,63]
***Acanthoscelides quadridentatus*** **(Bruchidae -Coleoptera)**	Attacks mature seeds	N/A	N/A	Attacks mature seeds and reduces seed rain, but has no impact on seed production.	[13,36,48,62]
***Acanthoscelides puniceus* (Bruchidae -Coleoptera)**	Attacks mature seeds	N/A	N/A	Attacks mature seeds.	[13,20,36]
***Chlamisus mimosae* (Chrysomelidae-Coleoptera)**	N/A	Attacks leaves and stems.			[20,36,62]
***Neurostrota gunniella* (Gracillariidae-Lepidoptera)**	N/A	Attacks pinnae and stems. Younger stems tend to have more larvae and are mostly concentrated at the edges of the stands. Reduces seed rain, radial canopy growth and seedling growth.			[20,36,51,62,64,65,66]
***Coelocephalapion aculeatum* (Curculionidae—Coleoptera)**	N/A	N/A	N/A	Attacks flowers and buds. Adults and larvae feed solely on flowers and flower buds.	[36,62,66,67]
***Coelocephalapion pigrae* (Curculionidae Coleoptera)**	N/A	Adults feed on leaves.		Larvae develop on flower buds and adults attack leaves and flowers	[20,68]
***Chalcodermus serripes* (Curculionidae-Coleoptera)**	Attack green seeds	Attacking developing tips.		Adults attack and feed on growing tips, flowers, and pods but oviposite only on seeds.	[36,62,69]
***Phloeospora mimosae-pigrae* (Coelomycetes- Deuteromycotina)**	N/A	Attacks leaves, stems and pods. Mostly attacks only primary and secondary leaf rachides and green stems.			[36,62,65]
***Diabole cubensis* L. (Pucciniaceae- Uredinales)**	N/A	Attacks plant leaves. Mostly the leaf pinnules.			[36,62,65]
***Sibinia fastigiata* (Curculionidae- Coleoptera)**	Attacks green seeds	N/A	N/A	Attacks green seeds and flowers.	[36]
***Malacorhinus irregularis* (Chrysomelidae -Coleoptera)**	Larvae feed on imbibed seeds	Larvae feed on seedlings and imbibe seeds while adults attack leaves, roots and nodules.			[36,70]
***Macaria pallidata* (Geometridae -Lepidoptera)**	N/A	Attacks leaves. Damage tends to concentrate on the top few recently emerged and fully developed leaves, and decreases with increasing leaf age.			[36,71]
***Leuciris fimbriaria* (Geometridae- Lepidoptera)**	N/A	Attacks leaves resulting in defoliation.			[36]

## Data Availability

Not applicable.
